# Fostering careers in aging: testing the impact of intergenerational learning on non-nursing students’ interest in China

**DOI:** 10.3389/fpsyg.2026.1773971

**Published:** 2026-03-19

**Authors:** Yang Wang, Huan Sun, Zhendong Wu

**Affiliations:** 1 Normal School of Vocational Techniques, Hubei University of Technology, Wuhan, China; 2 School of Law, Humanities and Sociology, Wuhan University of Technology, Wuhan, China

**Keywords:** ageism, aging career interest, China, inter-generational learning (IGL), non-nursing students

## Abstract

**Background:**

Confronted with a rapidly aging population and a severe shortage of aging-care professionals, China urgently needs to cultivate career interest among younger generations, particularly beyond traditional nursing pathways.

**Objective:**

To investigate the impact of a structured, theory-driven Intergenerational Learning (IGL) program, grounded in Contact Theory, on the aging-care career interest of non-nursing undergraduate students in China.

**Methods:**

Utilizing a pre-test/post-test design with an intervention group (*n* = 31) and a control group (*n* = 30), we measured outcomes using the *Willingness to Work with Elderly People Scale (WEPS)* and *the Fraboni Scale of Ageism (FSA),* complemented by post-intervention qualitative interviews.

**Results:**

Results indicate that the IGL program significantly enhanced students’ overall career interest compared to the control group, with notable improvements in the dimensions of Subjective Norm, Perceived Behavioral Control, and Intention. Path analysis revealed that the impact was partially mediated by a reduction in ageist attitudes. A compensatory effect was observed, whereby students with lower baseline interest benefited more from the intervention. Qualitative insights elucidated the mechanisms behind these quantitative findings, including the humanization of older adults and a crystallization of professional attitudes.

**Conclusion:**

The study suggests that a well-designed IGL intervention can be a promising strategy to address workforce gaps by fostering career interest among non-nursing students, although its effects vary across psychological dimensions and baseline characteristics.

## Introduction

The global increase in life expectancy has contributed to a continuous aging of the population. According to the *United Nations’ World Population Prospects 2024*, the global older population is projected to reach 994 million by 2030 and 1.6 billion by 2050 (United Nations, 2024). China is also confronting a severe demographic transition. With 216.76 million people aged 65 and above (15.38% of its total population) at the end of 2023, China is home to approximately one-quarter of the world’s older adults (National Bureau of Statistics of China, 2024). This demographic shift exerts immense pressure on social support systems, particularly in aging care. In China, about 35 million older adults lived with disabilities in 2023, a figure projected to rise to 46 million by 2035 (State Council of the People’s Republic of China, 2024; Cheng, 2024). Meeting the care needs of this population represents both a societal imperative and a significant familial burden, often resulting in substantial personal and economic costs for family caregivers (World Bank, 2023).

Concurrently, China faces a severe shortage of professionals in the aging-care sector. Current analyses indicate a workforce gap of 5.5 million in 2024, with projections suggesting this deficit could accumulate to nearly 59 million by 2030 (AgeClub, 2024). Operational realities further exacerbate the crisis: the caregiver-to-elderly ratio in China stands at approximately 1:10, far below the 1:4 standard recommended by the World Health Organization for adequate care. This workforce crisis unfolds against the backdrop of persistent challenges in youth employment among college graduates. Despite national policies like the *Employment Priority Strategy* (Communist Party of China Central Committee and State Council, 2024) actively encouraging graduates to enter the aging-care industry, structural barriers, including underfunded local services and the sector’s low social prestige, have limited their effectiveness, failing to bridge the workforce gap (Ministry of Civil Affairs and National Working Commission on Aging, 2024; Ministry of Finance, State Taxation Administration, and Ministry of Science and Technology of the People’s Republic of China, 2023; Liu et al., 2022; Ministry of Human Resources and Social Security and Ministry of Civil Affairs of the People’s Republic of China, 2019). Consequently, fewer than 400,000 graduates enter the sector annually, a figure critically insufficient to meet projected demand (Ministry of Civil Affairs, 2024).

This dual challenge, a critical shortage of aging-care professionals and underutilized graduate talent, necessitates innovative educational and intervention strategies. Enhancing collegiate interest in aging-care careers has thus emerged as a pressing research and policy imperative. In this context, Intergenerational Learning (IGL) presents a promising, theory-informed intervention (Ouyang and Zihe, 2022). IGL is defined as the collaborative participation of younger and older generations in purposeful, mutually beneficial activities to acquire knowledge, skills, and values (Fischer et al., 2014; Wang and Pang, 2020). Its potential efficacy is grounded in two key areas. First, Contact Theory posits that positive, structured interactions between groups can reduce prejudice and foster positive attitudes (Allport, 1954; Teater et al., 2021; Tseng et al., 2023). Meta-analytic evidence confirms that structured intergenerational contact can reduce ageism among young adults by over 30%, with effects sustained over time (Burnes et al., 2019; Gualano et al., 2018). Second, Experiential Learning Theory suggests that direct experience, as opposed to learning through stereotypes, can reshape perceptions and attitudes (Augustin and Freshman, 2016; Chonody, 2015). Applied to aging careers, positive intergenerational contact can improve attitudes toward older adults and increase interest in working with them (Gutheil et al., 2006). China’s cultural context, with its tradition of multigenerational family living, provides a natural foundation for such interventions, as prior exposure to older adults has been linked to greater caregiving willingness (Zhang et al., 2016).

However, significant gaps remain in both research and practice. Globally, studies on IGL’s impact on career interest have predominantly focused on nursing students (Garbrah et al., 2020; Okuyan et al., 2020; Zhang et al., 2016; Cozort, 2008), neglecting the vast potential of students from other disciplines who could fill diverse roles in the expanding aging-care ecosystem. In China, existing IGL initiatives have often been asymmetrically designed, primarily aiming to benefit older adults’ well-being while treating youth participation as auxiliary volunteerism (Febriani and Sanitioso, 2021). This approach risks overlooking IGL’s potential as a powerful pedagogical tool for shaping young people’s career attitudes and aspirations. Furthermore, prior intervention research suggests effect heterogeneity, where individuals with lower baseline interest may show greater gains—a compensatory effect (Harackiewicz et al., 2016; Roksa and Arum, 2011; Lundberg and Schreiner, 2004), yet this dimension remains underexplored in the context of IGL and aging careers.

Therefore, this study aims to address these gaps by investigating the impact of a structured IGL program on non-nursing undergraduate students’ interest in aging careers within the Chinese socio-cultural context. Specifically, our objectives are to: (1) Examine the impact of IGL on multidimensional aging-care career interest (attitudes, subjective norms, perceived behavioral control, and intentions); (2) Investigate the mediating role of reduced ageism (improved attitudes toward aging) in this relationship, drawing on Contact Theory and Stereotype Embodiment Theory (Levy, 2009); and (3) Explore potential differential impacts based on students’ initial levels of career interest. In this study, Contact Theory and Stereotype Embodiment Theory served primarily as guiding frameworks for designing the IGL intervention and formulating our hypotheses about the mediating role of reduced ageism. We operationalized their core propositions by structuring the IGL sessions to embody optimal contact conditions and by measuring ageism as a key intervening variable.

We hypothesize that:

*H1*: Participation in a structured IGL program will significantly increase students’ overall interest in aging-care careers.

*H2*: This impact will be mediated by a reduction in ageism (i.e., improved attitudes toward aging).

By answering these questions, this study seeks to contribute evidence on a scalable educational intervention that could simultaneously address two pressing social issues: preparing a future workforce for an aging society and creating meaningful career pathways for the younger generation.

## Literature review

### The challenge of workforce shortage and the potential of intergenerational learning

As delineated in the Introduction, China faces a critical shortage of professionals in the aging-care sector alongside underutilized graduate students. Cultivating interest in aging-related careers among university students, therefore, is not merely an educational issue but a socioeconomic imperative. However, research consistently shows that students across various disciplines, and particularly non-nursing majors, often hold low interest in pursuing such careers. This disinterest is frequently linked to pervasive negative attitudes and stereotypes about older adults and the work of caring for them (Feng et al., 2020; Neville, 2016). Consequently, educational interventions aimed at improving attitudes toward aging and older adults have been explored as a strategy to increase career interest.

Among these interventions, IGL has gained prominence. It is defined as the mutual exchange of knowledge, skills, and values between younger and older generations through structured, collaborative activities (Fischer et al., 2014). Its promise rests on a well-established theoretical foundation—Contact Theory (Allport, 1954). While Allport’s original work did not focus on aging, the core tenets of Contact Theory, that prejudice between groups can be reduced under conditions of equal status, common goals, intergroup cooperation, and institutional support, have been robustly applied and validated in intergenerational contexts (Burnes et al., 2019). Thus, when IGL programs are designed to incorporate these optimal contact conditions, they are theorized to facilitate positive intergenerational experiences.

### From positive contact to reduced ageism: the mediating role of ageist attitudes

The proposed causal chain begins with IGL facilitating positive intergenerational contact. Empirical meta-analyses support this link, confirming that structured intergenerational contact is an effective intervention for reducing ageism among younger people (Burnes et al., 2019). A key mechanism through which contact reduces ageism is by challenging and altering negative stereotypes.

This is where Stereotype Embodiment Theory (Levy, 2009) provides a crucial explanatory lens. Contrary to theories focusing solely on externally directed prejudice, Stereotype Embodiment Theory posits that age stereotypes, which are pervasive in culture and media, are internalized by individuals across the lifespan. These internalized stereotypes can operate unconsciously and influence self-perceptions and behaviors as one ages. For young adults, exposure to positive, counter-stereotypical interactions with older adults through IGL can disrupt this cycle of internalization. It provides direct, experiential evidence that contradicts generalized negative beliefs, thereby reducing the endorsement of ageist stereotypes (Ayalon et al., 2021). In other words, IGL does not merely aim to reduce outward prejudice but works by preventing the reinforcement of negative stereotypes that young people may have already begun to absorb from their socio-cultural environment.

### Linking improved attitudes to aging career interest

The final link in the logical chain connects improved attitudes to increased career interest. Research in career development and social psychology suggests that attitudes toward a social group significantly influence willingness to work with members of that group (Söylemez et al., 2022). Specifically in gerontology, studies have found a positive correlation between favorable attitudes toward older adults and higher interest in aging-related careers among students in health professions (Cheng et al., 2015; Söylemez et al., 2024; Cheng, 2021; McKinlay and Cowan, 2003). The rationale is that reduced ageism and more positive perceptions make the prospect of working with older adults seem more appealing, less daunting, and more socially valuable. Thus, by improving attitudes, IGL is positioned to indirectly enhance career interest.

Therefore, drawing on Contact Theory and Stereotype Embodiment Theory, we posit a core mediating mechanism: Well-structured IGL (incorporating contact theory conditions) facilitates positive, counter-stereotypical intergenerational experience, which directly challenges and reduces the internalization of negative age stereotypes (operationalized as lower scores on the Fraboni Scale of Ageism, *FSA*), thereby leading to increased interest in aging-care careers. In this model, reduced ageism (improved general attitudes toward older adults) is the key hypothesized mediator linking IGL to career interest.

### Gaps in the existing literature and the focus of the current study

Despite this theoretical rationale and supportive evidence, significant research gaps remain, which this study aims to address.

First, the evidence base is disproportionately focused on nursing and medical students (Algoso et al., 2016; Garbrah et al., 2020). While crucial, this focus overlooks the wide spectrum of professional roles needed in a comprehensive aging-care ecosystem (e.g., social work, community management, policy, technology). The impact of IGL on the career interest of non-nursing undergraduates remains largely unexplored.

Second, while the mediated pathway (IGL → attitudes → interest) is theoretically sound, few studies have empirically tested this full mediation model, especially within a controlled intervention design. Most research examines bivariate relationships or pre-post changes without elucidating the psychological mechanism.

Third, and critically, there is a paucity of such theory-driven intervention research in the Chinese cultural context. China presents a unique and pressing case: it faces the most rapid population aging globally, yet its aging care system is in transition from a family-based model, strongly influenced by filial piety, toward a more socialized one. This cultural context profoundly shapes attitudes. On one hand, the norm of filial respect may create a baseline of positive duty towards older adults within the family (Zhang et al., 2016). On the other hand, it may paradoxically devalue professional, paid aging care work, which can be seen as outsourcing a familial moral obligation, thus associating it with lower social prestige (Hui and Xu, 2023).

Furthermore, existing IGL programs in China have often been designed within this traditional paradigm, prioritizing the well-being of older adults and framing youth participation as volunteerism or filial duty practice, rather than as a pedagogical tool for modern career development in a socialized care system (Febriani and Sanitioso, 2021). Therefore, the impact of a reciprocal, theory-based IGL model, specifically aimed at shaping career interest in the emerging professional aging-care sector, within this distinct cultural setting is unknown and constitutes a key gap.

Finally, intervention effects are often heterogeneous. The concept of a compensatory effect, whereby individuals with lower initial interest or preparedness benefit more from an intervention, has been observed in educational psychology (Harackiewicz et al., 2016), but is rarely investigated in the context of IGL and aging career interest. Understanding for whom IGL works best is critical for targeted program design.

In summary, this study is designed to bridge these gaps by: (1) testing the impact of a structured IGL program on non-nursing Chinese undergraduates; (2) explicitly examining whether the impact on career interest is mediated by reduced ageism (improved attitudes); (3) conducting this investigation within the under-researched and socioculturally distinct context of China; and (4) exploring potential differential effects based on baseline career interest levels.

## Materials and methods

### Participants

#### Participant recruitment

The college students who participated in this research were sophomore students majoring in Public Administration, Sociology, and Social Work at a mid-tier provincial university in Wuhan, Hubei Province, enrolled in the researcher’s *Social Research Methods* course during the fall semester of 2024. This multidisciplinary sample aligns with the study’s focus on attracting non-nursing students to the broader aging-care sector. The modern aging-care ecosystem extends beyond clinical care to encompass policy design, community program management, social service delivery, and advocacy, areas where these disciplines provide foundational training (e.g., Cheng et al., 2015; Algoso et al., 2016). Students from these majors are thus a crucial talent pool for the diverse, non-clinical professional roles urgently needed to support comprehensive aging-care systems.

The older participants were recruited and organized through the directors of the Community Senior Activity Center, a common municipal facility in Chinese neighborhoods that provides recreational, social, and educational activities for older adults, promoting active aging and social integration. To facilitate their participation, most IGL sessions were held at this center, located within the community where the older adults resided.

#### Group allocation

The sample consisted of 85 participants (40 male, 45 female), with an average age of 21.2 years. Students were randomly assigned to either the intervention or control group by shuffling their student ID numbers and using a computer-generated random sequence. Of the 85 students enrolled in the study, 35 were assigned to the control group and 50 to the intervention group. This allocation ratio anticipated potential attrition over the course of the nearly semester-long intergenerational learning activities, accounting for possible withdrawal due to subjective or objective reasons during the extended intervention period. The older adult participants (*n* = 30) were randomly assigned to form eight mixed-age IGL groups, each comprising 4–5 older adults and 5–7 college students, who collaborated throughout the eight-session program.

#### Intervention and control conditions

Students in the intervention group participated in the community-based IGL program, which consisted of eight structured intergenerational sessions held bi-weekly over the 16-week semester. The control group consisted of students enrolled in the same *Social Research Methods* course who received identical classroom instruction on aging-related topics but did not participate in the community-based IGL sessions.

#### Course integration

During the 16-week intervention period (32 sessions total), the researcher integrated topics such as China’s aging trends, life expectancy among older adults, the health status of older adults and the current demands and challenges of aging care into the teaching of various social research methods. This approach aimed to enhance students’ understanding of and attention to issues related to older adults.

#### Final sample

Eligibility for the final intervention group sample was contingent upon participation in at least six intergenerational learning sessions. Of the 85 initially enrolled students, 61 completed both the pre-test and post-test surveys and were included in the final analysis. This final sample comprised 31 students in the intervention group (who attended at least six sessions) and 30 students in the control group.

#### Ethics approval statement

This study was approved by the Institutional Review Board of Hubei University of Technology. All procedures performed were in accordance with the ethical standards of the 1964 Declaration of Helsinki and its later amendments. Prior to data collection, all participants were provided with detailed information about the study’s purpose, procedures, potential risks and benefits, confidentiality safeguards, and their right to withdraw at any time without penalty. Written informed consent was obtained from all individual participants included in the study. To ensure confidentiality and anonymity, all questionnaires were completed anonymously, with students generating unique identification codes independently. This approach prevented instructors from linking responses to individual participants.

### Tests administered

Data were collected through a pre-test/post-test design using the *Willingness to Work with Elderly People Scale (WEPS) and the Fraboni Scale of Ageism (FSA).* Surveys were administered at the beginning (September 13, 2024) and end (January 11, 2025) of the 16-week semester.

Career interest was assessed using the *WEPS,* a psychometric instrument developed by Fadayevatan et al. (2018) to evaluate students’ willingness to engage in aging careers. The *WEPS* measures four dimensions of willingness to work with older adults: attitude, subjective norm, perceived behavioral control, and intention. Importantly, it avoids culture-specific references (e.g., religious or Western-centric concepts), which facilitates its adaptation in Chinese society. Previous studies in Turkey (Söylemez et al., 2022), Taiwan (Chi et al., 2016), and other Asian contexts have demonstrated the *WEPS’s* validity and reliability. For example, the Turkish adaptation (*WEPS-T*) showed excellent content validity (CVI = 1.00), strong internal consistency (*α* = 0.881), and stable test–retest reliability (*r* = 0.96, *p* < 0.001) (Söylemez et al., 2022). And the Taiwanese nursing students reported that *WEPS* scores correlated with positive attitudes toward older adults (*β* = 0.38, *p* < 0.001) and prior caregiving experience (*β* = 0.10, *p* = 0.005) (Chi et al., 2016). Similarly, the Turkish nursing students reported that *WEPS* scores were predicted by attitudes toward aging (*β* = 0.37, *p* < 0.001) and gerontology training (Akyol et al., 2024). These findings collectively support the *WEPS’s* cross-cultural stability in measuring willingness to work in aging careers across Asian societies.

Because the original *WEPS* was developed in English, we followed Brislin’s (1986) back-translation procedure to adapt it into Chinese, ensuring linguistic and conceptual equivalence. The process included four steps: (1) Forward Translation. Two bilingual researchers (fluent in English and Chinese) independently translated the original *WEPS* into Chinese; (2) Expert Panel Review: A committee of gerontology experts, educators, and linguists reviewed the translations for semantic, idiomatic, and conceptual equivalence. Discrepancies were resolved through discussion; (3) Back-Translation: A third translator, blinded to the original English version, re-translated the Chinese version back into English; (4) Cognitive Debriefing. A pilot test with 10 college students was conducted to assess item clarity and cultural appropriateness. Adjustments were made based on feedback. This rigorous process aligns with established best practices for cross-cultural scale adaptation (Söylemez et al., 2022) and preserves the original scale’s psychometric properties.

The *WEPS* demonstrates strong internal consistency, with a total score Cronbach’s alpha of 0.81. The scale comprises 20 items, organized into four distinct subscales: attitude (items 1–5), subjective norm (items 6–10), perceived behavioral control (items 11–15), and intention (items 16–20). Reliability analysis revealed acceptable Cronbach’s *α* values for these subscales: 0.54 (attitude), 0.57 (subjective norm), 0.73 (perceived behavioral control), and 0.84 (intention) (Fadayevatan et al., 2018). Participants rated their level of agreement with each statement on a 6-point Likert scale, ranging from “strongly disagree” (1) to “strongly agree” (6). To mitigate response bias, the *WEPS* includes reverse-coded items (items 2, 3, 4, and 17), which are scored inversely relative to their non-reversed counterparts. The total score on the *WEPS* ranges from a minimum of 20 to a maximum of 120, with higher mean scores indicating a stronger inclination toward pursuing aging careers (Fadayevatan et al., 2018). The specific items comprising the scale are detailed in Table 1.

**Table 1:** Items of *WEPS.*

Subdimensions	Items	Subdimensions	Items
Attitude	1 Working with the elderly is satisfying.	Perceived behavioral control	11 I achieve competences on elderly care.
	2 Care of elderly patients is a waste of financial resources.		12 I have professional competences on elderly care.
	3 Working with elderly patients is disappointing.		13 In the curriculum there is enough elderly care training.
	4 Working with the elderly is not a ***c*** areer.		14 I have the skill of working with the elderly.
	5 Working with the elderly is a highly useful experience.		15 I have necessary capabilities to provide end-of-life care.
Subjective Norm	6 Caring for the elderly is a human duty.	Intention	16 One of my career priorities after graduation is elderly care.
	7 My culture encourages me to work with the elderly.		17 I will never consider aging care a job.
	8 There is enough encouragement to work with the elderly.		18 I would like to work in community elderly care after graduation.
	9 My professors advise me to consider a career on elderly care.		19 I prefer to work with the elderly after graduation.
	10 Working with the elderly is socially valuable.		20 I will certainly choose to work with the elderly after graduation.

The Cronbach’s alpha values observed in our study (Attitude: 0.55–0.78, Subjective Norm: 0.66–0.79, Perceived Control: 0.62–0.89, Intention: 0.64–0.85) are generally consistent with those reported in the original validation by Fadayevatan et al. (2018). Some subscales yielded moderate alpha values, such as Attitude in the post-test of the Intervention group (0.55) and Intention in the pretest of the control group (0.64), which is not uncommon for short subscales comprising five items each. According to Streiner (2003), such values are considered acceptable in exploratory research, as alpha is sensitive to scale length. Moreover, the stronger reliability observed for the full scale (*α* = 0.82–0.90) and for the Perceived Control subscale (α = 0.62–0.89) further supports the overall reliability of the measure (see Table 2).

**Table 2:** Cronbach’s alpha scores for the *WEPS.*

Scale	Intervention group		Control group			
	Pretest	Post-test	Pretest	Post-test	Number of items	Item
Total *WEPS*	0.89	0.90	0.82	0.83	20	1–20
Attitude	0.67	0.55	0.78	0.59	5	1–5
Subjective norm	0.79	0.75	0.66	0.68	5	6–10
Perceived control	0.89	0.85	0.62	0.79	5	11–15
Intention	0.65	0.85	0.64	0.69	5	16–20

The assessment of college students’ ageism was conducted utilizing the *FSA*, a psychometrically validated tool developed by Fraboni et al. (1990) to systematically evaluate attitudes toward older adults. The Chinese version of *FSA* has been culturally adapted and validated for use in mainland China, demonstrating satisfactory reliability (Cronbach’s α = 0.81, ICC = 0.87) and validity (CVI = 0.93; three-factor structure confirmed) among medical students (Fan et al., 2020). More recently, the *FSA* was further validated in a study involving 392 long-term caregivers in Chinese nursing homes, where it exhibited strong internal consistency (*α* = 0.86) and test–retest reliability (ICC = 0.87), with exploratory and confirmatory factor analyses supporting its three-factor structure (explaining 43.95% of total variance) (Li et al., 2024). These findings collectively confirm the *FSA’s* cross-population applicability in measuring ageism among both healthcare professionals and students, highlighting its potential to identify ageist attitudes that may negatively impact aging care quality and caregiver well-being.

The 29-item *FSA* employs a Likert-scale format to systematically assess ageist tendencies among college students. Higher composite scores reflect stronger ageist attitudes directed toward older individuals. Participants were required to rate their degree of agreement with each statement using a 4-point Likert scale, anchored by “strongly disagree” (1) at one end and “strongly agree” (4) at the other. To address potential response bias, the scale incorporates reverse-coded items (items 8, 12, 14, and 21–24), which are inversely scored in comparison to their non-reversed counterparts. The *FSA* yields a total score range spanning from a minimum of 29 to a maximum of 116, with higher cumulative scores signifying more pronounced manifestations of ageist attitudes.

In the foundational work by Fraboni et al. (1990) with Canadian college students, the scale demonstrated high internal consistency (*α* = 0.86), good test–retest reliability (*r* = 0.83 over 2 weeks), and convergent validity with Palmore’s Facts on Aging Quiz (*r* = −0.42, *p* < 0.01). The *FSA* has established psychometric credentials across cultures. The scale’s validity is further evidenced by its negative correlation with gerontological knowledge measures and ability to detect ageism reduction in educational interventions.

Our data showed comparable psychometric performance: cronbach’s α = 0.87 (pre-test), 0.90 (post-test) of Intervention group, item-total correlations ranged 0.21–0.65; cronbach’s α = 0.87 (pre-test), 0.84 (post-test) of control group, item-total correlations ranged 0.23–0.78. Beyond its original validation (α = 0.86; Fraboni et al., 1990), our implementation showed strong reliability and item coherence.

## Procedure

### Program design rationale

The IGL program in this study was explicitly designed around Allport’s (1954) Contact Theory and its later extensions by Pettigrew (1998) to create optimal conditions for positive intergenerational contact. Unlike traditional service-learning, which often positions youth as unidirectional helpers, this IGL model was built on reciprocity, co-learning, and mutual value creation (Peterat and Mayersith, 2008; Pinazo-Hernandis, 2011). We structured each session to satisfy four key contact conditions:

Equal-status: interaction-activities were framed as collaborative tasks where both generations contributed expertise;
Common goals: each session had a shared, tangible outcome (e.g., a craft product, a health plan, a community proposal);
Intergroup cooperation: tasks required active collaboration and interdependence;
Institutional support: the program was formally embedded in a university course and partnered with a community senior center, providing legitimacy and resources.

The program also integrated career scaffolding by exposing students to aging care roles through site visits, role modeling, and reflective discussions about professional pathways, a component rarely included in generic service-learning (Teater, 2018).

### Program dosage and context

The intervention consisted of 8 structured sessions held bi-weekly over a 16-week semester. This design represents a feasible, intensive exposure integrated into an existing *Social Research Methods* curriculum, testing the potential of a theory-driven IGL model within the practical constraints of Chinese higher education.

### Detailed activity schedule

The core components of the eight IGL sessions, and their linkage to aging-care career exposure are described (see Table 3).

**Table 3:** Structured IGL activities based on contact theory.

Session	Themes	Core activities	Contact-theory conditions met
1	Chongyang festival: cultural co-creation	Joint handicraft production: Groups create paper-cutting, painting, or origami works themed on “respect for older adults”; Paired life-story introductions: Students and older partners share personal backgrounds.	Equal status (both are creators / storytellers); Common goal (complete a craft piece); Cooperation (mutual guidance)
2	Red songs and social debates	Red-song guessing game: Lyric completion, title identification, and intergenerational teaching of classic revolutionary songs; Structured debate: Discussion on “low birthrate and aging society”	Common goal (prepare a mini-performance); Institutional support (venue, host); Emotional engagement
3	Digital inclusion workshop	One-on-one smartphone tutoring: Students teach WeChat, mobile payment, health-tracking apps; Co-creation of “Digital Safety Guide”: Older adults share real-life scam experiences; students add technical tips.	Role reversal (youth as experts); Mutual benefit (skills exchange); Cooperative task (produce a guide)
4	Health promotion across generations	Tai Chi / Baduanjin demonstration: Older adults lead students in traditional exercises; Health-dialogue circle: Students share modern nutrition/exercise science; older adults contribute lived experience.	Equal status (both are health-knowledge holders); Common goal (design a joint health plan); Collaborative activity
5	Aging-care career immersion	Visit to community aging-care center: Staff explain daily operations, care models, career paths; Role-playing scenarios: Students and older adults simulate caregiver-resident, social-worker-family interactions.	Positive exemplars (young staff as role models); Cooperative reflection (debrief together); Institutional support (site access)
6	Community co-design workshop	Walk-through assessment: Joint inspection of community spaces for accessibility issues; Design prototyping: Groups propose improvements and create simple models/posters.	Common goal (submit a proposal to community); Interdependence (combine observational + technical skills); Cooperative problem-solving
7	Life-story interviewing and legacy project	Semi-structured life-history interviews: Students conduct in-depth conversations about older adults’ life journeys; Producing a “Intergenerational Life-Story Booklet”: Compile photos, quotes, reflections into a digital/print memoir.	Personalized contact (beyond group stereotypes); Emotional bonding; Shared creative product
8	Capstone showcase and career dialogue	Exhibition of all session outputs (crafts, health plans, community designs, storybooks); Panel discussion: Invited speakers (care-sector employees, graduates) discuss aging-industry opportunities.	Public recognition (celebration of achievements); Linking contact to broader social roles Institutional endorsement

Session 1: Chongyang Festival Co-creation. Activities included collaborative handicraft making (e.g., paper-cutting, painting) themed on older respect and initial paired life-story sharing. Career Exposure: Students experienced how cultural and artistic activities are integral to psychosocial care and community-based aging services.

Session 2: Red Songs and Social Debates. This session featured a red-song guessing and teaching game, followed by a structured intergenerational debate on topics like low birthrate and family values. Career Exposure: It demonstrated the role of communication, life review, and facilitating dialogue across generations, which are key skills in gerontological social work and community engagement.

Session 3: Digital Inclusion Workshop. Students provided one-on-one tutoring to older adults on using smartphones and common apps (WeChat, mobile payment), while co-creating a digital safety guide with older adults’ input. Career Exposure: This direct “digital caregiving” experience highlighted a growing service demand in aging care and the teaching/coaching role of care professionals.

Session 4: Health Promotion Exchange. Older adults led traditional exercise practices (e.g., Tai Chi), and both generations engaged in a health dialogue circle sharing knowledge on nutrition and wellness. Career Exposure: Students observed the integration of traditional and modern health management approaches, a core component of holistic aging care and health promotion professions.

Session 5: Aging-Care Career Immersion. The group visited a community aging-care center, interacted with staff, and participated in role-playing scenarios simulating caregiver-resident interactions. Career Exposure: This direct site visit and role-play provided concrete exposure to the care environment, daily routines, and the multifaceted roles of care professionals, reducing abstraction and stigma.

Session 6: Community Co-design Workshop. Students and older adults jointly assessed community spaces for accessibility and collaboratively designed proposals for age-friendly improvements. Career Exposure: The activity introduced concepts of environmental gerontology and community planning, showcasing careers focused on creating supportive living environments for older adults.

Session 7: Life-Story Interviewing and Legacy Project. Students conducted in-depth life-history interviews with their older partners and co-created a “Life-Story Booklet” integrating photos and narratives. Career Exposure: This exercise provided hands-on practice in narrative methods and ethical documentation, core competencies in geriatric counseling, social work, and biographical care.

Session 8: Capstone Showcase and Career Dialogue. The final session featured an exhibition of all session outputs and a panel discussion with professionals from the aging-care sector discussing career pathways. Career Exposure: It connected the experiential learning to real-world career opportunities, provided networking prospects, and socially validated aging-care professions through interaction with role models.

After each IGL session, students conducted brief one-on-one interviews with the older participants in their group to deepen understanding of the older adults’ life experiences, needs, and perspectives, thereby reinforcing the reciprocal learning process and enriching the qualitative data collection.

### Statistical procedures

All statistical analyses were performed using SPSS (Version 27.0) and Mplus (Version 8.3).

#### Preliminary analyses

Prior to parametric testing, we verified all statistical assumptions and found that all parametric tests met required assumptions: Shapiro–Wilk tests confirmed normality of score differences (Intervention group: *W* = 0.989, *p* = 0.985; Control group: *W* = 0.974, *p* = 0.862), no outliers exceeded ±3.29 SDs, and Welch’s t-test addressed variance heterogeneity (post-test SDs: Intervention group = 1.46 vs. Control group = 3.47). Subsequently, descriptive statistical analyses, such as means and standard deviations, were employed to examine the item-total score and item-subdimensions.

#### Baseline comparisons

Independent samples *t*-tests were conducted to compare baseline (pretest) scores between the intervention and control groups. These analyses confirmed no statistically significant differences in total *WEPS* scores (intervention: *M* = 69.77, *SD* = 12.07; control: *M* = 64.07, *SD* = 11.36), *t*(59) = 1.92, *p* = 0.060, nor in any subscale scores (all *p* > 0.05), establishing baseline equivalence between groups (see Tables 4, 5).

**Table 4:** Comparisons of *WEPS* total score and subscale scores between pretest and post-test for the intervention group (*N* = 31).

Scale	Pretest		Post-test					Effect size	Observed Power
	*M*	*SD*	*M*	*SD*	GAP	*t*	*p*		
Total *WEPS*	69.77	12.07	72.74	12.27	2.97	1.66	0.107	0.30	0.34
Attitude	20.87	1.50	20.55	1.48	−0.32	−0.94	0.353	0.17	0.14
Subjective norm	21.71	4.55	22.78	4.15	1.06	1.45	0.158	0.26	0.28
Perceived behavioral control	13.97	5.77	14	5.03	0.03	0.04	0.971	0.01	0.05
Intention	13.23	4.09	15.42	5.16	2.19	2.99	0.005	0.54	0.82

**Table 5:** Comparisons of *WEPS* total score and subscale scores between pretest and post-test for the control group (*N* = 30).

Scale	Pretest		Post-test					Effect size	Observed Power
	*M*	*SD*	*M*	*SD*	GAP	*t*	*p*		
Total *WEPS*	64.07	11.36	63.07	14.83	−1.00	−0.47	0.641	−0.076	0.06
Attitude	21.47	4.77	22.30	5.32	0.83	0.70	0.488	0.164	0.14
Subjective norm	19.03	4.92	18.07	4.50	0.97	−0.37	0.353	−0.206	0.06
Perceived behavioral control	11.27	3.85	11.37	4.62	0.01	0.09	0.924	0.020	0.05
Intention	12.30	3.57	11.33	4.31	0.97	−1.28	0.210	−0.246	0.23

①According to Cohen’s standards: 0.2 ≤ |*d*| < 0.5: Small effect size; 0.5 ≤ |*d*| < 0.8: Medium effect size; |*d*| ≥ 0.8: Large effect size; Power < 0.80 indicates insufficient sensitivity to detect small-to-medium effects given current sample sizes.

#### Within-group changes

Paired samples *t*-tests were used to examine changes from pretest to posttest within each group separately.

#### Statistical analyses

A series of one-way ANCOVAs were conducted to test the intervention effect on post-test outcomes (total *WEPS* and four subscales) while controlling for baseline differences, with group assignment entered as the fixed factor and the corresponding pretest score as the covariate. Multiple regression analyses were performed to examine the predictive relationships between IGL participation, ageism, and career interest, with post-test *WEPS* scores entered as dependent variables and group assignment and post-test *FSA* scores as predictors.

**Figure 1:**
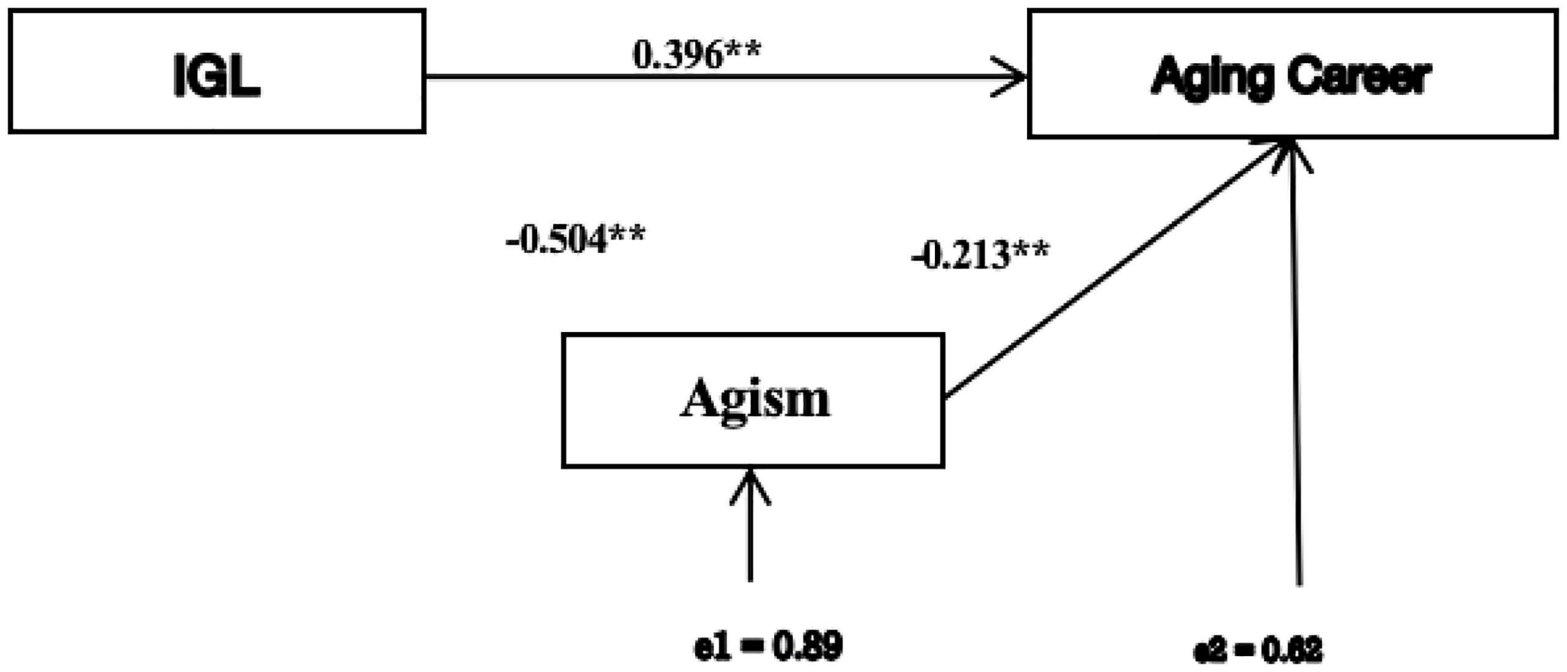
Path analysis.

**Figure 2:**
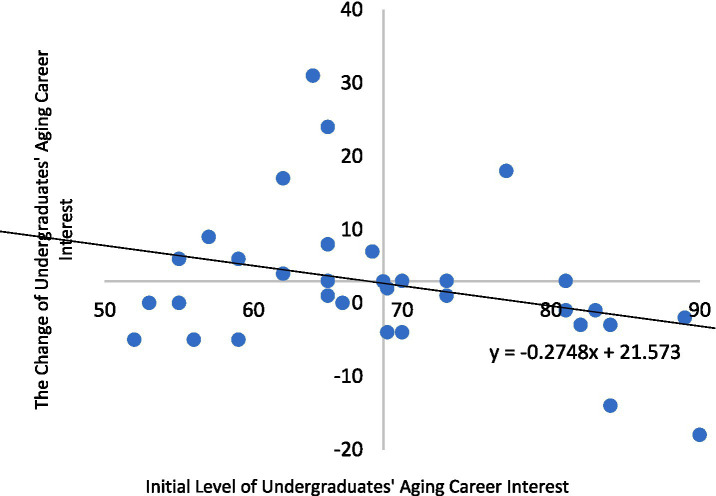
Two dimensional four quadrant distribution of college students based on initial interest scores and magnitude of change of *WEPS.* ① In the graph, the horizontal axis represents the initial interest scores of college students in aging careers, while the vertical axis represents the magnitude of change in their interest. ② The origin point (69.77, 2.94) represents the means of initial interest scores and the magnitude of change, respectively. ③ In the graph, the number of college students distributed across the first, second, third, and fourth quadrants is 4, 10, 7, and 10, respectively, accounting for 12.90, 32.26, 22.58, and 32.26% of the total sample. The mean and standard deviation of the number of students across the four quadrants are 7.75 and 2.87, respectively.

To investigate whether ageism mediated the effect of IGL on career interest, a path analysis was conducted using Mplus, testing direct paths from group to *WEPS* and from group to *FSA*, and a path from *FSA* to *WEPS*. Indirect effects were calculated using the product-of-coefficients method, and model fit was assessed using *χ*^2^/df, CFI, RMSEA, and SRMR (see Figure 1). To explore heterogeneous treatment effects, change scores were calculated for intervention group, a Pearson correlation was conducted between baseline *WEPS* scores and change scores, and participants were stratified into quadrants to visualize the pattern of change (see Figure 2).

### Supplementary qualitative data collection

To gain a deeper, contextualized understanding of the intervention’s impact beyond quantitative measures, semi-structured interviews were conducted with 15 purposefully selected participants from the Intervention Group approximately 1 week after the post-test survey. Participants were purposefully selected according to criteria designed to maximize variation. This was based on their quantitative change scores on the total *WEPS* (categorized as high positive, minimal, or negative change), and their baseline interest level, in order to capture a wide spectrum of experiences and perspectives.

The interview protocol focused on exploring their lived experiences, perceived changes, and program evaluations during the IGL activities. Core questions included: 1. “Can you describe a particularly memorable moment or interaction from the IGL sessions?” 2. “How have your thoughts or feelings about older adults as a group changed after participating in this program?” 3. “Before and after the program, how did you view careers in aging care? What factors influence your interest in such careers?” 4. “What aspects of the IGL program were most valuable or impactful to you? What could be improved?”

Each interview lasted 30–45 min, was audio-recorded, and later transcribed verbatim for thematic analysis. This mixed-methods approach was employed to triangulate the quantitative findings and provide rich explanatory insights.

## Results

### Interest in aging career

As demonstrated in Table 4, participation in IGL activities contributed to a measurable improvement in overall career interest among the intervention group students, evidenced by a 2.97-point increase in the total *WEPS* score, though this change did not reach statistical significance (*p* = 0.107, *d* = 0.30). Subscale analyses revealed differential patterns. No significant changes were observed in the Attitude (*p* = 0.353), Subjective Norm (*p* = 0.158), or Perceived Behavioral Control (*p* = 0.971) subscales. However, the Intention subscale showed a significant increase of 2.19 points (*p* = 0.005, *d* = 0.54). The observed power for the Intention subscale was sufficient (0.82), whereas power for the remaining subscales and the total score was low (0.05–0.34). These findings suggest that IGL participation had a pronounced and statistically meaningful impact on students’ intention to pursue aging-related careers, but did not yield significant improvements in overall career interest, attitude, subjective norm, or perceived behavioral control. This highlights that while IGL can effectively enhance career intention, a more integrated approach may be needed to positively influence the broader psychological dimensions of career decision-making.

For the control group, no significant changes were observed between pretest and posttest in total or subscale scores (*p* > 0.05), with effect sizes ranging from negligible to small (Cohen’s *d* = −0.246 to 0.164) and low observed power (0.05–0.23) (see Table 5). These results indicate that integrating aging-related content into the standard curriculum—without structured IGL activities—did not significantly enhance students’ career interest in aging-related professions.

A comparison of post-test outcomes between the intervention and control groups revealed significant differential effects of IGL participation (see Table 6). The intervention group achieved a significantly higher total *WEPS* score than the control group (*M*_diff_ = 9.68, *p* = 0.007, *d* = 0.71). Analysis of subscales showed that the intervention group scored significantly higher on Subjective Norm (*M*_diff_ = 4.70, *p* = 0.001, *d* = 1.09), Perceived Behavioral Control (*M*_diff_ = 2.63, *p* = 0.038, *d* = 0.54), and Intention (*M*_diff_ = 4.09, *p* = 0.0014, *d* = 0.86). Notably, the intervention group scored lower on Attitude, though this difference was not statistically significant (*M*_diff_ = −1.77, *p* = 0.08, *d* = 0.45). Post-hoc power analysis indicated adequate to high power (0.60–0.99) for detecting these significant effects, except for Attitude (power = 0.47).

**Table 6:** Comparisons of post-test *WEPS* total score and subscale scores between the intervention group and control group (*N* = 31, 30).

Scale	Intervention group		Control group					Effect size	Observed power
	*M*	*SD*	*M*	*SD*	GAP	*t*	*p*		
Total *WEPS*	72.74	12.27	63.07	14.83	9.68	2.78	0.007	0.71	0.88
Attitude	20.55	1.48	22.30	5.32	−1.75	- 1.77	0.080	0.45	0.47
Subjective norm	22.78	4.15	18.07	4.50	4.70	4.26	0.001	1.09	0.99
Perceived behavioral control	14.00	5.03	11.37	4.62	2.63	2.13	0.038	0.54	0.60
Intention	15.42	5.16	11.33	4.31	4.09	3.35	0.001	0.86	0.96

### Differential intervention effects by baseline interest levels

To explore heterogeneous treatment effects, we stratified participants by baseline interest levels. Analysis of change scores (*M* = 2.94, mode = 3; range: −18 to 31) revealed substantial variability in student responses to the IGL program, with some showing increased interest while others exhibited declines.

Students were categorized by initial interest levels using the pretest mean (69.77) as a cutoff. Among those with strong initial interest (> 69.77), 19.36% showed positive change post-intervention, while 32.26% declined. Conversely, 38.71% of students with weak initial interest (< 68.74) demonstrated improvement, with only 9.68% showing reduced interest. Quadrant analysis (Figure 2) revealed an inverse correlation (*r* = −0.28), indicating that higher initial interest tended to correspond with smaller gains. Only 35.48% of students followed the expected pattern: 12.90% in Q1 (high interest/large gains) and 22.58% in Q3 (low interest/small changes). The majority (64.52%) exhibited atypical responses: 32.26% in Q2 (low interest/large gains) and 32.26% in Q4 (high interest/small changes).

This inverse relationship aligns with the *compensation effect*, suggesting that the IGL program disproportionately benefited students with initially lower interest levels. The findings highlight the program’s compensatory potential, particularly for those less predisposed to aging-related careers, while revealing limited impact on students who began with stronger interest. Given the modest sample size, this observed pattern of differential impacts is preliminary and warrants investigation in larger samples.

This finding may convey an encouraging signal, as the survey results suggest that Chinese college students generally exhibit a low interest in pursuing aging careers. The *WEPS* total score ranges from 20 to 120 points, with each of the four subscales (comprising 5 items each) ranging from 5 to 30 points. Using 60% of the maximum as a reference, the corresponding benchmarks are 72 points for the total score and 18 points for each subscale. The average total scores of students in the intervention group were 69.77 (pretest) and 72.74 (posttest), remaining near this total score reference point. At pretest, mean scores for Subjective Norm (21.71), Perceived Behavioral Control (13.97), and Intention (13.23) fell below the subscale reference point of 18 points. Following the IGL activities, a significant increase was observed only for the Intention subscale (posttest *M* = 15.42, *p* = 0.005, *d* = 0.54), bringing it closer to but still below the reference point. And changes in the other subscales, including Subjective Norm and Perceived Behavioral Control, were not statistically significant.

### Inter-generational learning enhances aging career interest by improving attitudes toward aging

To test the hypothesized theoretical mechanism that IGL enhances career interest partly by reducing ageist attitudes, we conducted a path analysis. This model examines whether the association of IGL participation and *WEPS* scores is mediated by changes in *FSA* scores, representing reduced ageism.

#### Baseline equivalence of *FSA* score

Independent samples t-tests showed no significant difference in attitudes toward aging between the intervention group (*M* = 57.44, *SD* = 9.84) and the control group (*M* = 61.40, *SD* = 11.33) at baseline (*t* = 1.50, *p* = 0.139), confirming the initial comparability of the two groups before the intervention. The effect size was small (Cohen’s *d* = 0.39), and the observed statistical power was low (0.35), further supporting the conclusion of baseline comparability between the two groups prior to the intervention.

#### Post-intervention between-group differences in *FSA* score

After the intervention, the control group reported significantly higher post-test scores (*M* = 59.23, *SD* = 13.81) than the intervention group (*M* = 51.77, *SD* = 10.76), with a mean difference of 7.46 (*t* = 2.36, *p* = 0.021). This between-group difference was associated with a medium effect size (Cohen’s *d* = 0.61) and an acceptable level of observed power (0.65), suggesting that the community-based intervention may have effectively mitigated negative attitudes toward aging in the intervention group.

#### Within-group changes over time of *FSA* score

Paired samples t-tests revealed distinct patterns of change within each group from pre- to post-test. The intervention group showed a statistically significant reduction in scores, indicating improved attitudes, with pre-test *M* = 57.16 (*SD* = 10.03) decreasing to post-test *M* = 51.78 (*SD* = 10.76), a mean decrease of 5.39, *t* = −3.07, *p* = 0.0045. This change corresponded to a medium effect size (Cohen’s *d* = 0.55) and high observed power (0.85). In contrast, the control group showed no significant change over time, with pre-test *M* = 61.04 (*SD* = 11.33) and post-test *M* = 59.23 (*SD* = 13.81), a mean decrease of 2.17, *t* = −0.57, *p* = 0.571, accompanied by a small effect size (*d* = 0.10) and very low observed power (0.08).

Based on multiple regression and ANCOVA results (Table 7), participation in the IGL program showed a positive but non-significant predictive impact on overall interest in aging careers (overall interest: *F*[1, 46] = 1.83, *p* = 0.183, η^2^ = 0.038). When analyzed by subdimensions, however, the influence of IGL was differential: it significantly strengthened subjective norms (*F*[1, 46] = 8.20, *p* = 0.006, η^2^ = 0.116), exerted a positive but non-significant impact on perceived behavioral control, and led to a significant decline in attitudes toward pursuing aging careers (*F*[1, 46] = 9.80, *p* = 0.003, η^2^ = 0.164). These findings suggest that IGL exerts a multifaceted influence on interest in aging careers—potentially enhancing social normative perceptions while undermining personal attitudes. The results were consistent across analytical approaches, though the modest sample size (*N* = 61) warrants replication in larger studies.

**Table 7:** Multiple Regression (N = 61).

Scale	Coef.	Std. Err.	*t*	*p* > |*t*|	*R*-squared
Total *WEPS*	0.325	0.166	1.96	0.055	0.484
Attitude	−0.344	0.201	−1.65	0.103	0.080
Subjective norm	0.670	0.192	3.50	0.001	0.368
Perceived behavioral control	0.296	0.227	1.30	0.199	0.231
Intention	−0.311	0.135	−2.31	0.001	0.426

Separate regression models were run for each dependent variable (total score and subscales). Multicollinearity diagnostics indicated Variance Inflation Factor (VIF) values for all predictors were below 2.0.

As shown in Figure 1, regression analyses of pre- and post-test data revealed a significant negative correlation between ageism and career interest, suggesting that more negative perceptions of older adults were associated with lower interest in pursuing aging careers. The model demonstrated acceptable fit to the data (*χ*^2^/df = 2.22, CFI = 0.96, RMSEA = 0.07, SRMR = 0.03, supporting further interpretation of individual paths. Path analysis results (see Figure 1) further suggested that the direct impact of IGL activities on career interest was significant (Coef. = 0.396, *Z* = −2.25, *p* = 0.028). However, the indirect impact of IGL, mediated through improved attitudes toward aging, was calculated as (−0.504) × (−0.213) = 0.107. These findings suggest that both direct and attitude-mediated pathways contribute to IGL’s influence, highlighting the dual importance of the intervention itself and its role in addressing attitudinal barriers to promote interest in aging professions. It is important to note that the path analysis was conducted with a total sample of *N* = 61. While the model fit indices were acceptable (*χ*^2^/df = 2.22, CFI = 0.96, RMSEA = 0.07, SRMR = 0.03), parameter estimates from path models with small samples can be unstable. Therefore, the mediation results should be interpreted as “preliminary and exploratory”, and the parameter estimates interpreted with caution, requiring replication with larger samples for robust confirmation.

### Thematic insights from participant interviews

To gain a deeper, contextualized understanding of the intervention’s impact and to elucidate the mechanisms underlying the quantitative findings, semi-structured interviews were conducted with 15 purposefully selected participants from the Intervention Group. Thematic analysis (Braun and Clarke, 2006) of the interview transcripts yielded three salient themes that directly explain and enrich the quantitative results presented in Sections 4.1 to 4.3. These themes clarify the observed “attitude paradox”elucidate the compensatory effect, and provide a nuanced account of how IGL reshaped perceptions (see Table 8).

**Table 8:** Representative quotations from participant interviews illustrating key themes.

Participant ID	Theme	Quotation (Translated from Chinese)
P04	Theme 1: From Stereotype to Individuality	“I used to think of ‘older adults’ as one group that needed help. Now I see Mr. Li, who taught me paper-cutting, and Grandma Wang, who was so competitive in the quiz. They’re all different people with their own personalities.”
P09	Theme 1: From Stereotype to Individuality	“The media always shows older people as weak or sick. But in the debate, they had such strong opinions about family and society. It made me rethink what I see online.”
P11	Theme 2: The “Compensatory Effect” Explained	“I went in with no thought of this as a career, but seeing how we could actually help and connect… it made me reconsider. Maybe there’s something here I had not thought about.”
P02	Theme 2: The “Compensatory Effect” Explained	“I was surprised by how much I enjoyed just talking to them and hearing their stories. It did not feel like ‘work’ at all.”
P07	Theme 2: The “Compensatory Effect” Explained	“It was fun, but it felt more like a community activity. I wish we had talked more about what a real career in this field looks like, the challenges and the specialties.”
P13	Theme 3: Clarifying the “Attitude Paradox”	“I have more respect for the work now, but I also see how hard it really is—emotionally and physically. It’s not just a simple ‘good thing to do’ anymore. It’s a real, tough job.”
P15	Theme 3: Clarifying the “Attitude Paradox”	“Before, I just thought ‘helping the elderly’ was good. Now I think about it: Do I have the patience every day? Can I handle the sad parts? My attitude is more… complicated now.”

#### Theme 1: From stereotype to individuality: humanizing older adults

This theme provides a qualitative mechanism for the significant reduction in ageism (*FSA* scores) observed in the Intervention Group, illustrating how contact disrupted internalized stereotypes. Participants consistently described a shift from viewing older adults as a homogeneous, stereotyped group to appreciating them as diverse individuals. Direct interactions challenged preconceptions of frailty, passivity, and technological incompetence. As one participant noted,*“I used to think of ‘older adults’ as one group that needed help. Now I see Mr. Li, who taught me paper-cutting, and Grandma Wang, who was so competitive in the quiz. They’re all different people with their own personalities.”*

#### Theme 2: The compensatory effect explained: divergent pathways of engagement

This divergence explains why the IGL intervention acted as a more potent catalyst for those with lower initial interest, directly supporting the quantitative finding of a compensatory effect. Interview data offered a clear rationale for the differential gains in career interest. Students with initially low baseline interest frequently expressed surprise and discovery, focusing on the unexpected enjoyment and relational rewards of the interaction (e.g., “*I went in with no thought of this as a career. It made me reconsider”*). In contrast, students with higher pre-existing interest evaluated the experience through a more critical, professional lens, often desiring more substantive content on career pathways and skills, which the short-term program lacked (e.g., *“I wish we had talked more about what a real career in this field looks like*”).

#### Theme 3: clarifying the “Attitude Paradox”: nuancing professional perceptions

The interviews provided crucial insight into the counterintuitive decline in the Attitude subscale of the *WEPS*. Participants indicated that their evaluation of an“aging-care career” evolved from a vague, socially desirable ideal to a more concrete and critically assessed professional domain. Post-intervention, they considered the practical, emotional, and physical demands of the work more acutely. One participant articulated this shift:*“I have more respect for the work now, but I also see how hard it really is. It’s not just a simple ‘good thing to do’ anymore. It’s a real, tough job.”* This process of attitude crystallization, resulting in a more realistic, sometimes guarded appraisal, likely explains the lower scores on attitudinal items that presuppose an uncomplicated positive view, thereby clarifying the “attitude paradox” observed in the quantitative data.

## Discussion

This study provides valuable insights into the impact of IGL on undergraduate students’ interest in aging-related careers in China, a country facing dual challenges of a rapidly aging population and high youth unemployment. The findings suggest that IGL can enhance students’ interest in aging careers, particularly through improvements in subjective norms and intentions, as measured by the *WEPS*. However, the results also highlight the complex and multifaceted nature of IGL’s influence, with significant variations in its impact based on students’ initial interest levels and the specific dimensions of career interest.

The study makes several important theoretical contributions.

First, it extends the literature on IGL by demonstrating its potential to foster career interest among non-nursing students, a population that has been largely overlooked in previous research. This finding resonates with Experiential Learning Theory and Contact Theory (Allport, 1954), positing that hands-on, interactive experiences can reshape perceptions and attitudes, thereby cultivating career interest (Levy, 2009; Ayalon et al., 2021). Specifically, the IGL program in this study was explicitly designed around Allport’s (1954) Contact Theory principles—emphasizing equal status, common goals, intergroup cooperation, and institutional support. This structured design provided participants with direct, positive, and counter-stereotypical interactions. The qualitative interview data vividly illustrate how students moved from viewing older adults as a homogeneous group to appreciating their unique individuality and capabilities, offering concrete evidence for the mechanisms through which contact reduces ageism.

Second, the study introduces the concept of a compensatory effect within IGL research. The finding that students with lower baseline interest derived greater benefits from the IGL activities challenges conventional assumptions of a linear relationship between initial interest and intervention outcomes. It suggests that experiential learning programs may function differentially for individuals with varying baseline engagement levels. The qualitative interviews (Theme 2) provide a nuanced explanation: students with low initial interest focused on the unexpected enjoyment and relational rewards of the interaction, whereas those with higher pre-existing interest evaluated the experience through a more critical, professional lens, desiring more substantive career-related content. This finding aligns with theories of career construction and self-efficacy, which emphasize the dynamic nature of career development and the role of experiential learning in reshaping aspirations (Powers et al., 2013).

Third, the study investigates the mediating role of attitudes in the IGL—career interest relationship, revealing nuanced complexities. While supporting the theoretical proposition that improved attitudes can serve as a pathway, our results also highlight differential impacts across dimensions of career interest. ANCOVA results indicated that IGL significantly strengthened subjective norms but led to a significant decline in attitude scores as measured by the *WEPS.* The qualitative data (Theme 3) are crucial for interpreting this *“attitude paradox.”* They reveal a process of attitude crystallization, whereby students’ evaluations evolved from a vague, socially desirable ideal to a more concrete, realistic, and critically assessed appraisal of the profession. This suggests that the lower scores on attitudinal items, which presuppose an uncomplicated positive view, reflect a more mature and informed understanding rather than a negative shift, thereby clarifying the paradoxical quantitative finding.

From a practical perspective, the findings offer implications for designing and implementing IGL programs, though these require further validation through larger-scale, longitudinal studies.

First, the study highlights the need for IGL activities to be tailored to address the diverse needs of students. For students with lower initial interest, IGL can act as an effective “interest primer.” For those with higher baseline interest, programs should provide more advanced, specialized content on career development to sustain and deepen their engagement. Designers should consider stratified or modular strategies.

Second, the study highlights the importance of integrating IGL into formal curricula and academic programs. The IGL program in this study was not an isolated volunteering activity but a carefully designed component embedded within a *Social Research Methods course,* ensuring structure, reciprocity, and academic relevance. While short-term activities can improve certain interest dimensions, more systematic, semester-spanning integrated courses—combining intergenerational contact, skill-building, career exploration, and reflective practice are needed for lasting impact.

Third, enhancing college students’ interest in aging-related careers requires a collaborative effort involving educational institutions, government, and society. The findings serve as a reminder that IGL activities alone, as an extracurricular and informal interactive approach, are insufficient to fundamentally alter college students’ career interest. Liam and Jing (2023) found that standalone educational programs had limited long-term impact on career choices without systemic support. Universities should integrate IGL objectives into course goals, provide experiential learning opportunities beyond the classroom, and include career guidance, similar to the multidisciplinary approach advocated by Jussara et al. (2022). Additionally, governmental policy advocacy, industry engagement, and societal recognition through mass media are essential to position aging-care services as a socially recognized and highly professional career. For instance, comprehensive gerontology programs or courses could be established to embed the understanding and appreciation of older adults into academic instruction, positioning aging-care services as a socially recognized and highly professional career. While some studies suggest that formal education can significantly shift career interests (Ann, 2022), our focus on informal IGL activities aligns with prior work highlighting the limitations of extracurricular interventions without broader structural support (Maley et al., 2017). This discrepancy may stem from differences in intervention intensity or cultural context, warranting further research.

### Limitations and future directions

Despite its contributions, this study has several limitations.

First and foremost, the sample comprised students from Public Administration, Sociology, and Social Work—disciplines relevant to non-clinical roles in aging care. However, this focus excludes other potential non-nursing majors (e.g., business, engineering, design) and does not account for heterogeneity in career aspirations even within the selected majors. Therefore, our findings should be generalized with caution. Future research should include a broader range of disciplines to test the transferability of the IGL impact and examine how disciplinary backgrounds moderate its impact.

Second, the use of the *WEPS* in this non-nursing context presents several limitations. While validated in nursing studies, its applicability here is constrained by potential cultural mismatch, items concerning “professional autonomy” or “salary expectations” may carry different connotations in collectivist societies, and by its original development for nursing students, which may not fully capture the perceptions of students from public administration, sociology, or social work, nor reflect the broader range of aging-care roles (e.g., policy, management).

Moreover, the moderate internal consistency of the Attitude and Subjective Norm subscales (*α* = 0.55–0.79) may have limited our ability to detect significant within-group changes in the intervention group, potentially obscuring smaller true effects. Future research should develop or adapt scales with stronger cross-cultural validity and psychometric robustness for diverse non-nursing populations.

Third, the sample size, while adequate for detecting main intervention effects(Sara et al., 2023), is limited for the more complex statistical models employed, such as path analysis and subgroup comparisons examining differential effects. The parameter estimates in the path analysis, while theoretically coherent, may be unstable. The statistical power for some tests (e.g., pre-post comparisons on certain subscales) was also limited. These quantitative findings related to the mediation pathway and the compensatory effect should be viewed as exploratory, preliminary and hypothesis-generating, requiring replication with larger samples.

Fourth, regarding intervention dosage and methodological constraints, the IGL program lasted one semester (8 sessions). While intensive, longer or more sustained interventions might yield deeper impacts. Furthermore, the quasi-experimental design, though methodologically rigorous within practical constraints, limits strong inference compared to a fully randomized trial. Specifically, unmeasured confounding cannot be fully ruled out; therefore, the observed effects should be interpreted as promising evidence of the intervention’s potential impact, pending confirmation by future randomized controlled trials.

Fifth, although not statistically significant, the baseline difference in total *WEPS* scores between the intervention and control groups approached significance (*p* = 0.06), with the intervention group showing marginally higher initial interest. This trend suggests a potential pre-existing difference that could have partially contributed to the post-intervention findings. Future studies should employ more rigorous randomization or matching procedures to ensure strict baseline equivalence.

Despite these limitations, the mixed-methods approach—complementing quantitative data with rich qualitative interviews, strengthened the study by providing mechanistic explanations for the observed patterns. Future research should adopt longitudinal, mixed-methods designs with larger, multi-institutional samples and more robust interventions to overcome these constraints and confirm the relationships identified here.

## Conclusion

In conclusion, this study suggests that a structured, theory-driven IGL program designed around Contact Theory (Allport, 1954) principles, holds promise for addressing the dual challenges of an aging society and youth career development. It can foster interest in aging careers by facilitating positive intergenerational contact, improving attitudes toward aging (albeit with complex manifestations in scale scores), and strengthening subjective norms and intentions. The qualitative interview data were instrumental in elucidating the underlying mechanisms, explaining both the compensatory effect and the attitude paradox. However, the effects of IGL are not uniform and are moderated by students’ baseline interest, program design, and socio-cultural context. It is crucial to note that, due to sample size limitations, the robustness of some quantitative findings, including the path analysis, requires further verification.

To maximize the potential of IGL, future efforts should focus on tailored program design, deeper curricular integration, and building a collaborative ecosystem of support. Through the synergy of educational innovation and social policy, we can cultivate a diverse pipeline of young professionals equipped with both competency and compassion for an aging world. This study provides an empirical and theoretical foundation for such endeavors.

## Data Availability

The raw data supporting the conclusions of this article will be made available by the authors, without undue reservation.
